# Event and Comment

**Published:** 1918-02

**Authors:** 


### EVENT AND COMMENT

**Save the Deciduous Teeth**

Dr. C. N. Johnson, in an editorial in the *Dental Review*, makes the following comment on the value of children's teeth, which needs careful attention of most dentists, as well as parents who do not attach sufficient importance to the children's first teeth.

"In the minds of many dentists and of most parents there seems to be the sole idea of relieving the child of present disability without thought of the permanent injury which may result. It is not only the matter of impairing the mastication for the time being by the extraction of these teeth, but of the far greater injury in an entirely different direction. Orthodontists have repeatedly called attention to the serious disarrangement of articulation in the permanent set by the premature loss of the deciduous teeth, and this is particularly true of the molars. If these teeth are lost long in advance of the eruption of the bicuspids which are to take their place, the first permanent molars, which are usually erupted four or five years before the bicuspids, are almost certain to drift forward and narrow the space intended for the bicuspids. This results in irregularity of the permanent teeth, in some instances to such an extent that the second bicuspid is caught between the first bicuspid and first permanent molar and never allowed to erupt. The havoc wrought in some mouths by the premature loss of even one deciduous molar is so serious that dentists should take great pains to instruct parents on the necessity of saving the teeth of their children.

Ordinarily these teeth are amenable to treatment even after they are decayed sufficiently to involve the pulp, and in many instances even when they have gone further than this and developed an abscess. Usually these abscesses readily yield to treatment, but of course if they do not the tooth must come out. The chief lesson to learn is that the child's deciduous teeth should be examined regularly and kept in such condition that no abscess develops. The practice of dentistry is becoming more and more a matter of educating the public, and in no department of our service is it quite so important as in the one under consideration.

In those cases where the loss of the deciduous molars seems inevitable on account of long neglect, the case should be referred to an orthodontist to have an appliance adjusted to maintain the normal space till the bicuspids are fully in place. This will usually save the child from much subsequent trouble in having the teeth regulated later in life after irreparable damage has been done."

Dr. Lourie says it is just as important to keep the deciduous denture intact so that the child can masticate efficiently as it is to attempt to provide for the permanent denture by expanding the deciduous arch by orthodontic procedure. We believe it is more important.

**American Institute of Dental Teachers**

The twenty-fifth annual meeting of this most interesting organization was held in Pittsburg, as the guest of the University of Pittsburg School of Dentistry, January 29 to 31, 1918. Representatives from forty dental colleges were in attendance. Boston, California, Minnesota, Tennessee, Georgia, two Canadian colleges, and all the colleges of the Eastern, Western and Middle States sent one delegate at least, and several colleges had five or six delegates in attendance. The program was interesting and instructive and filled the entire time of the three-days session. The proceedings of this convention are intensely interesting and would be appreciated by the practitioner as well as the teacher. It is probable that the proceedings will be published this year in the *Journal of the National Dental Association.* If so we are confident they will be read with benefit by the practitioners who have never attended the meetings of this organization. Some of the brightest men in our profession are teachers and naturally they are good talkers, and the discussions of papers read before this body are worth while. The Pittsburg meeting was well attended in spite of the difficulties of travel, and the proceedings were of unusual interest because of the war complications and the necessity for arranging suitable courses for students who are preparing to enter service, as well as special courses for practitioners who are enlisting and need reviewing and preparation for the special war demands in surgery and other war dentistry. One evening session was devoted to a banquet given by the University of Pittsburg. The speeches were all of a patriotic character, and it would be difficult to describe the enthusiasm of the occasion. The speeches were the best we have heard, and the earnestness and eloquence of the speakers was fully appreciated by everyone.

In the earlier days of this organization only two subjects were considered in its programs, and exhibits of a technical character were the principal features. Then there was considerable rivalry of the colleges in the matter of technical exhibits and the showing of teaching appliances. At this meeting there were quite a number of excellent exhibits, mostly of new or unique methods of illustrating class or laboratory means for imparting instruction. There has been a constant tendency for several years to enlarge the literary program and to give less time to exhibits of a technical character. The changing of the course by the additional year of instruction has made the demand for more attention being given to the theoretical rather than less to the technical studies. The addition of more instruction in clinical and technical studies has also increased the time as well as the subjects to be taught. It is true that some of the added subjects can not all be taught by the usual technical methods. There seems to be a general tendency to give greater emphasis to the instruction which will increase the student's scientific knowledge and also give him a broader culture in medical science as it is related to dental knowledge and practice. All the teachers at this meeting seemed impressed with the idea that a closer relation with medicine was demanded and the new curriculum must keep this in mind when making up the work schedule for the four-year course.

**Rochester Dental Dispensary Report** The dispensary was opened for clinical work October 15, 1917, and the following report to February, 1918, shows how rapidly the work is developing. There are now twenty-five graduate dentists at work in the infirmary, and more patients apply than can be given treatment.

In October 853 patients were treated; 736 paid the fee of five cents.

In November 3,185 patients were treated; 2,770 paid the fee of five cents.

In December 3,910 patients were treated; 3,236 paid the fee of five cents.

In January 5,104 patients were treated; 3,687 paid the fee of five cents.

The following are the more important treatments:

October: Treatments, 1,503; roots treated, 528; fillings, 916; extractions, 272.

November: Treatments, 4,329; roots treated, 1,807; fillings, 2,521; extractions, 515.

December: Treatments, 4,741; roots treated, 1,938; fillings, 2,793; extractions, 604.

January: Treatments, 6,264; roots treated, 2,653; fillings, 3,611; extractions, 1,077.

Besides this, the thirty-nine dental hygienists working in the schools under the direction of the graduate instructors have inspected and given prophylaxis treatment to nearly all the public and parochial schools of the city. It is hoped that when the work is fully organized the schools will be inspected and given treatment at least once every year. Careful records are being made which ought to be of great scientific value in the future. This is surely a great work.

